# MFF-ClassificationNet: CNN-Transformer Hybrid with Multi-Feature Fusion for Breast Cancer Histopathology Classification

**DOI:** 10.3390/bios15110718

**Published:** 2025-10-29

**Authors:** Xiaoli Wang, Guowei Wang, Luhan Li, Hua Zou, Junpeng Cui

**Affiliations:** 1 Electronics Information Engineering College, Changchun University, Changchun 130022, China; 230401158@mails.ccu.edu.cn (G.W.); 230401173@mails.ccu.edu.cn (H.Z.); 240401193@mails.ccu.edu.cn (J.C.); 2 School of Electrical Automation and Information Engineering, Tianjin University, Tianjin 300072, China; lulhanli2005@tju.edu.cn

**Keywords:** breast cancer, multi-feature fusion, histopathological images, transformer, attention mechanism

## Abstract

Breast cancer is one of the most prevalent malignant tumors among women worldwide, underscoring the urgent need for early and accurate diagnosis to reduce mortality. To address this, A Multi-Feature Fusion Classification Network (MFF-ClassificationNet) is proposed for breast histopathological image classification. The network adopts a two-branch parallel architecture, where a convolutional neural network captures local details and a Transformer models global dependencies. Their features are deeply integrated through a Multi-Feature Fusion module, which incorporates a Convolutional Block Attention Module—Squeeze and Excitation (CBAM-SE) fusion block combining convolutional block attention, squeeze-and-excitation mechanisms, and a residual inverted multilayer perceptron to enhance fine-grained feature representation and category-specific lesion characterization. Experimental evaluations on the BreakHis dataset achieved accuracies of 98.30%, 97.62%, 98.81%, and 96.07% at magnifications of 40×, 100×, 200×, and 400×, respectively, while an accuracy of 97.50% was obtained on the BACH dataset. These results confirm that integrating local and global features significantly strengthens the model’s ability to capture multi-scale and context-aware information, leading to superior classification performance. Overall, MFF-ClassificationNet surpasses conventional single-path approaches and provides a robust, generalizable framework for advancing computer-aided diagnosis of breast cancer.

## 1. Introduction

Breast cancer is a leading malignant tumor that poses a significant threat to women’s health globally, ranking as the primary cause of mortality among female malignancies. In China, the incidence and mortality rates of breast cancer account for 11.7% and 9.2% of global cases, respectively, placing it among the highest worldwide [1,2]. According to the World Health Organization (WHO), this number is projected to rise to 19.3 million by 2025 [3]. Recent studies have increasingly focused on the molecular mechanisms and drug resistance of breast cancer. Research by Pavitra et al. [4] and Raju et al. [5] has elucidated relevant mechanisms, highlighting the importance of histopathological image analysis in early diagnosis and targeted therapy. In clinical practice, pathologists primarily rely on ultrasound images, X-ray images, and histopathology images (HI) to distinguish breast cancer subtypes. Among these, histopathology images contain rich tissue and cellular information and play a critical role in breast cancer diagnosis. Histopathologic diagnosis is widely regarded as the gold standard for definitive breast cancer diagnosis [6], involving the acquisition of tissue sections from suspicious lesions and examination under microscopes at different magnifications [7] to clearly visualize tissue structures, providing pathologists with the most reliable diagnostic evidence. Currently, clinical diagnosis mainly depends on pathologists’ manual analysis of histopathology images, a process that is time-consuming, labor-intensive, and influenced by individual experience and expertise, introducing a degree of subjectivity. Therefore, developing computer-aided diagnosis (CAD) tools, integrating computer vision and deep learning technologies, can significantly improve diagnostic accuracy, reduce misdiagnosis, and ultimately save more lives.

The rapid advancement of deep learning and computer-aided systems has significantly improved cancer screening technologies [8,9]. In breast histopathology, considerable efforts have focused on distinguishing benign from malignant cases [10,11], though achieving robust performance remains challenging. Early computer-aided diagnosis (CAD) relied on handcrafted features (e.g., texture descriptors) and traditional classifiers such as SVM, K-NN, and MLP. With the rise of medical image analysis, CNNs have become the dominant approach due to their superior accuracy and robustness [12]. Bardou et al. [13] demonstrated that CNN-based deep features outperform handcrafted ones, and architectures like VGGNet [14], ResNet [15], and DenseNet [16] have shown promising results. Joseph et al. [17] proposed a CNN architecture that efficiently extracts rich, scale-invariant features. Spanhol et al. [18] developed a modified AlexNet using sliding window and random sampling strategies for lesion detection, achieving 89.6% accuracy. Wang et al. [19] introduced DBLCNN, enhancing MobileNet with transfer learning to improve feature representation, reduce model complexity, and maintain high recognition performance.

In contrast, Soham Chattopadhyay et al. [20] introduced the Dense Residual Dual-shuffle Attention Network (DRDA-Net), incorporating Residual Dual-shuffle Attention Blocks (RDAB) and dense connections. This architecture emphasizes the learning of fine-grained tissue structures through attention-guided feature refinement and effectively mitigates overfitting and vanishing gradient issues. Its advanced attention design sets it apart from traditional CNN variants, making it a more robust and adaptive solution for complex histopathological image classification tasks.

The Transformer [21], originally developed for sequence-to-sequence prediction in NLP, has gained significant popularity in computer vision. The Vision Transformer (ViT) model, introduced by Dosovitskiy et al. [22], splits an image into patches with positional embeddings, forming a sequence of tokens. These tokens pass through Transformer blocks to extract parametric vectors that serve as visual representations, effectively capturing complex spatial relationships and long-range dependencies for global semantic modeling. However, ViT struggles to capture fine-grained local spatial details, limiting feature extraction thoroughness. To address this, Yuan et al. [23] proposed using CNN feature maps as input tokens to better represent neighborhood features. Nevertheless, these approaches still treat images as one-dimensional token sequences, neglecting local inductive biases, which affects convergence speed and overall performance.

CNNs, owing to their inherent inductive biases such as locality and translation equivariance, are highly effective at capturing fine-grained local spatial features. However, they often struggle to model long-range dependencies and holistic contextual information. In contrast, Transformer-based models, with their powerful self-attention mechanisms, excel at representing global relationships but tend to overlook local structural details and generally require large-scale datasets for optimal performance. To fully leverage the complementary strengths of CNNs and Transformers in the medical domain, this paper proposes a Multi-Feature Fusion Classification Network (MFF-ClassificationNet). The network aims to enhance classification accuracy and robustness by combining the local feature extraction capability of CNNs with the global modeling power of Transformers. It employs a parallel-branch architecture, comprising a Detail Enhancement Module and a Global Feature Module for deep feature extraction. Additionally, A Convolutional Block Attention Module—Squeeze and Excitation (CBAM-SE) Fusion block (CSF) is embedded within the Multi-Feature Fusion (MFF) module to integrate local and global attention features. Specifically, the CSF integrates the Squeeze-and-Excitation (SE) attention module, the Convolutional Block Attention Module (CBAM), and the Inverted Residual Multi-Layer Perceptron (IRMLP). Through this fusion mechanism, fine-grained local details and global features at multiple scales are effectively combined, enabling comprehensive feature extraction and adaptive feature integration. The proposed model is evaluated on the publicly available BreakHis dataset [8], with extensive experiments confirming its superior classification performance. It should be noted that the experiments and evaluations in this study were conducted exclusively on breast cancer histopathology images, and the model’s applicability to other types of cancers remains to be explored in future research. Furthermore, its generalization capability is validated on the BACH dataset [24].

The main contributions of this work are summarized as follows:

(1) To address the problem that traditional methods in breast histopathological image classification struggle to simultaneously capture local details and global semantic information, resulting in insufficient feature representation, a novel Multi-Feature Fusion Classification Network (MFF-ClassificationNet) was designed, which uses a parallel dual-branch architecture to enhance local details while modeling global features.
(2) To address the limitation of existing methods in multi-scale feature fusion, which restricts the model’s ability to comprehensively utilize information at different levels, a CBAM-SE Fusion (CSF) block was introduced, integrating SE, CBAM, and IRMLP modules to achieve adaptive multi-scale feature fusion.
(3) Extensive experiments conducted on BreakHis and BACH datasets demonstrate the superior performance of the proposed method in terms of classification accuracy and generalization capability.

The paper is organized as follows: The Introduction highlights the importance of breast cancer diagnosis and the potential of CAD. Section 2 describes the datasets, Section 3 details the MFF-ClassificationNet architecture, Section 4 presents experimental results and comparisons, and Section 5 concludes the study.

## 2. Dataset Used

### 2.1. BreakHis Dataset

**Figure 1:**
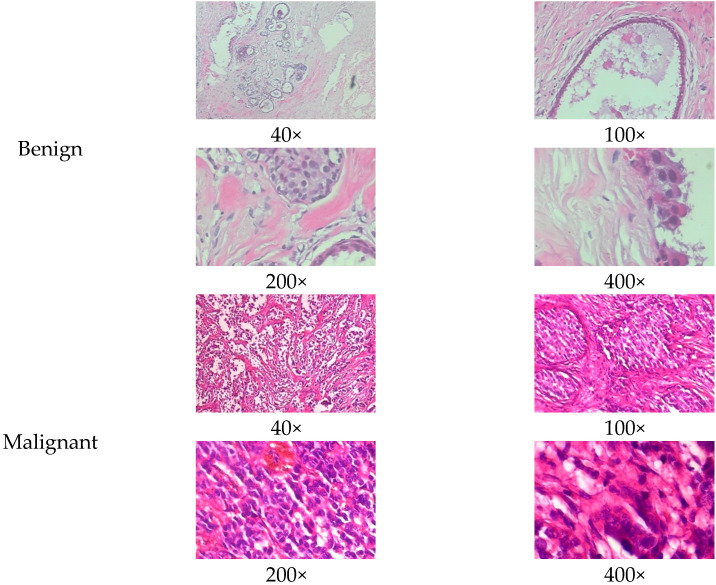
Histopathology sample images from the BreakHis dataset.

The BreakHis dataset [8] is a widely utilized public resource comprising microscopic biopsy images of benign and malignant breast tissues. Collected by the P&D Laboratory in Brazil, all patient data within the dataset has been anonymized. It features breast tissue samples obtained via surgical open biopsy, with expert pathologists providing the corresponding labels. The dataset consists of 7909 images, each with a resolution of 700 × 460 pixels, and is categorized into four magnification levels: 40×, 100×, 200×, and 400×. As shown in Table 1, each magnification level contains approximately 2000 images. The dataset is further partitioned into training, validation, and test sets for model evaluation. Despite its value in medical imaging research, BreakHis poses two significant challenges for deep learning models. Firstly, its relatively small size increases the risk of overfitting. Secondly, a notable class imbalance exists at each magnification level, with malignant samples being nearly twice as numerous as benign ones, which can affect model performance. As illustrated in Figure 1, representative histopathological images of benign and malignant breast tissues are shown for each magnification level (40×, 100×, 200×, and 400×), providing a visual overview of the dataset.

### 2.2. BACH Dataset

**Figure 2:**
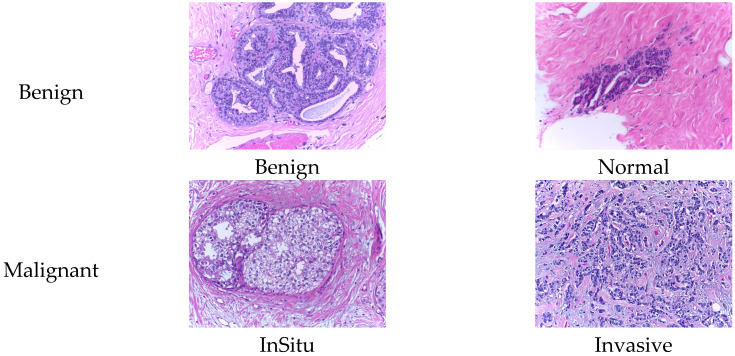
Histopathology sample images from the BACH dataset.

The BACH dataset [24] was introduced as part of the 2018 ICIAR (International Conference on Image Analysis and Recognition) competition. All images were acquired under consistent conditions using a 200× magnification and were meticulously annotated by two expert pathologists. This dataset comprises 400 Hematoxylin-Eosin stained breast histopathology images, classified into two primary categories: Non-Carcinoma (Benign) and Carcinoma (Malignant). Additionally, these are further divided into four subtypes: Normal, Benign, In Situ Carcinoma, and Invasive Carcinoma. The specific distribution of the dataset is presented in Table 2. As shown in Figure 2, representative histopathological images of the four subtypes in the BACH dataset—Normal, Benign, In Situ Carcinoma, and Invasive Carcinoma—are presented, providing a visual overview of the dataset.

## 3. Proposed Method

**Figure 3:**
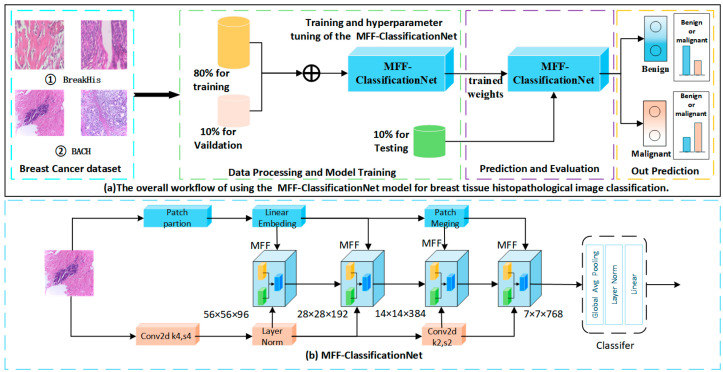
The workflow of the study and a schematic diagram of the MFF-ClassificationNet model. (**a**) The overall workflow of breast tissue histopathological image classification using the MFF-ClassificationNet model. (**b**) The overall architecture of the MFF-ClassificationNet model.

The proposed MFF-ClassificationNet network employs a multi-feature fusion architecture, which includes a detail enhancement path, a global feature path, and a CSF module, enabling effective multi-scale feature extraction and improving classification performance. The workflow of this study is illustrated in Figure 3a and consists of the following key steps: First, a breast tissue histopathological image dataset containing both benign and malignant samples is used for model training and evaluation. The dataset is split into 80% for training, 10% for validation, and 10% for testing. During the training phase, the data is first preprocessed by resizing all images to a fixed resolution to ensure uniform input dimensions for the model, facilitating consistent feature extraction and effective training. Following preprocessing, the MFF-ClassificationNet model is trained, and its performance is further optimized through hyperparameter tuning. After training, the optimized model with its best-trained weights is used for prediction and evaluation. Ultimately, the model can classify histopathological images as benign or malignant and output the corresponding classification results. By incorporating a multi-feature fusion approach, this study aims to enhance the classification performance of breast tissue histopathological images, providing clinicians with a more accurate and reliable auxiliary diagnostic tool, thereby improving the accuracy and effectiveness of early breast cancer detection and treatment.

### 3.1. Multi-Feature Fusion Network

To improve the accuracy of breast histopathology image classification, this paper proposes MFF-ClassificationNet, a parallel-path network that integrates local details with global features for efficient multi-scale feature extraction. As shown in Figure 3b, the model adopts a dual-branch design: one path divides the image into patches embedded into a high-dimensional space, while the other extracts initial features using Conv2D and layer normalization. Both paths feed into four Multi-Feature Fusion (MFF) modules at different stages, where dual-path features are fused to generate hierarchical feature maps (56 × 56 × 96, 28 × 28 × 192, 14 × 14 × 384, 7 × 7 × 768). The fused features are then compressed via Global Average Pooling, normalized, and classified through a fully connected layer. MFF-ClassificationNet combines the local feature extraction capability of CNNs with the global modeling capacity of Transformers to enable multi-scale feature representation for histopathological image analysis.

### 3.2. Multi-Feature Fusion Module

**Figure 4:**
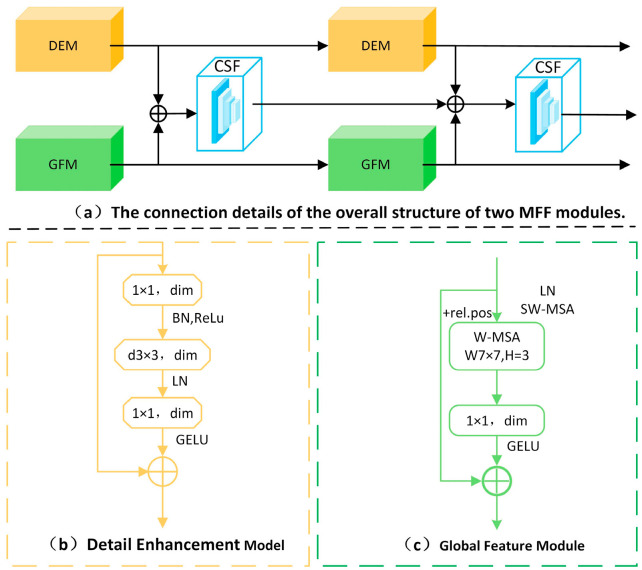
(**a**) Structural diagram of two MFF connections; (**b**) Detail Enhancement Module (DEM) detail diagram; (**c**) Global Feature Module (GFM) detail diagram.

The structure of the MFF module is depicted in Figure 4a. It comprises two parallel branches, specifically designed to extract both fine-grained details and global semantic features from breast tissue histopathological images. The detail enhancement branch is centered around a ResNet block, which serves as its core structure. By adjusting channel numbers and integrating features, this branch learns richer and more discriminative representations of local details. The global feature branch utilizes a patching module to segment the image, treating each 4 × 4 neighboring pixel region as a patch while flattening the channel dimensions. The merged features are then processed through a linear embedding layer, doubling the number of output channels. Following this, a global feature block performs feature transformations to capture the image’s overall semantic representation. Information extracted from both branches is further refined and integrated within the CSF, which plays a crucial role in enhancing and combining detailed and global features at each stage, while also connecting them with outputs from the previous layer. This design facilitates multi-level, multi-scale feature fusion, effectively combining fine-grained local details with broader semantic context for downstream classification tasks.

#### 3.2.1. Detail Enhancement Module

The complexity of medical histopathological image analysis is influenced by multiple factors, leading to potential similarities between different categories. Therefore, detailed features are crucial in medical image analysis. The detail enhancement block, as shown in Figure 4b, is a modified version of the ResNet Block. First, a 1 × 1 convolution layer adjusts the feature map’s channel count by performing a linear combination of different channels for feature fusion. Batch Normalization (BN) is applied during training to normalize input distributions, helping to accelerate network convergence, while the ReLU activation function mitigates the vanishing gradient problem in deep networks. The 3 × 3 convolution layers, BN, and ReLU in the ResNet Block are replaced with 3 × 3 dependency convolutions. This change reduces the network’s computational complexity (Floating Point Operations per Second, FLOP). Next, Layer Normalization (LN) is applied, followed by a fully connected (linear) layer and the GELU activation function, which facilitates cross-channel information interaction. Lastly, the extracted local features are fed into the CSF. This process is mathematically formulated in Equation (1):

(1) $$D_{i}=D^{1\times 1}(LN(D^{d3\times 3}BN({conv}^{1*1}(D_{i-1}))))+D_{i-1}$$

where $D_{i}$ represents the output features of the detail enhancement block, ${conv}^{1*1}$ represents a convolution operation with a 1 × 1 kernel, LN signifies a Layer Normalization operation, ${conv}^{d3*3}$ denotes a depthwise convolution with a 3 × 3 kernel, and BN corresponds to a Batch Normalization operation.

#### 3.2.2. Global Feature Module

Given the significant variations within the same category, capturing global semantic information is essential for accurate analysis and diagnosis. The global feature block, depicted in Figure 4c, is designed to address this requirement. The key component in global feature extraction is the Window-based Multi-Head Self-Attention (W-MSA) mechanism, which is adapted from the Swin Transformer [25]. Compared to the traditional Multi-Head Self-Attention (MSA) module in Transformer architectures, W-MSA offers a more efficient approach by significantly reducing computational complexity. Initially, the input feature map is partitioned into multiple fixed-size windows, with self-attention computed independently within each window. Next, the feature vectors within each window are divided into multiple heads, each computing its own attention weights. Finally, the self-attention outputs are combined back into the original feature map shape. The computational complexity of W-MSA can be expressed in Equation (2):

(2) $$\Omega (W-MSA)={4hwC}^{2}+2M^{2}hwC$$

where h represents the height of the feature map, w denotes the width, C indicates the depth, and M refers to the size of each window.

During the global feature extraction phase, Layer Normalization (LayerNorm) is applied to the feature map to maintain consistent feature scaling, helping to prevent issues like gradient explosion and vanishing gradients for more stable training. The normalized feature map is then processed through the W-MSA module, followed by a linear layer with the GELU activation function. GELU enables a smooth transition from zero to linearity, allowing the model to effectively handle a diverse range of input values. To preserve information flow, a residual connection is incorporated after each module by adding the output back to the input. Additionally, relative position bias is introduced to enhance the model’s understanding of spatial relationships. Since W-MSA restricts information exchange to within individual windows, the Shifted Window Multi-Head Self-Attention (SW-MSA) mechanism, also adopted from the Swin Transformer [25], is employed to shift the windows in a predefined manner, thereby creating overlapping regions with adjacent windows. This strategy enhances local feature extraction while maintaining global context. The mathematical formulation of this process is provided in Equation (3):

(3) $$G_{i}={conv}^{1\times 1}(SW-MSA(LN\{W-MSA(LN(G_{i-1}))+G_{i-1}\}))+g_{i}$$

where $G_{i}$ and $g_{i}$ represent the output features from the SW-MSA and W-MSA modules in the global feature block, respectively. ${conv}^{1*1}$ refers to the 1 × 1 convolution operation. LN denotes the LayerNorm operation.

The effective fusion of features from different scales in each branch presents a new challenge. To address this, the CSF module is introduced, which integrates the extracted detailed information and global features into the CSF for fusion.

### 3.3. CSF Module

**Figure 5:**
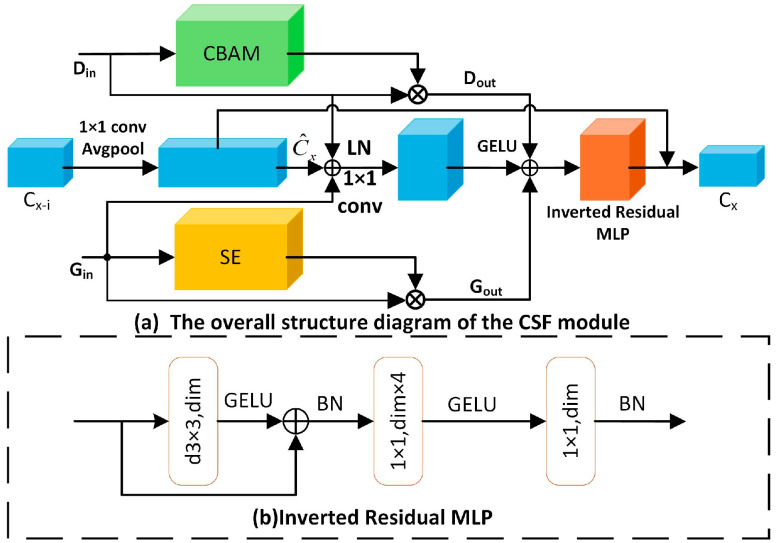
CSF module design: (**a**) Overall structure of CSF module; (**b**) Detailed structure of IRMLP.

The CSF feature fusion block dynamically combines detailed information, global features, and the fused output from the previous layer across different levels based on the input features. The overall structure is illustrated in Figure 5a. Here, Din denotes the feature matrix produced by the detail enhancement module, Gin represents the feature matrix from the global feature module, C_x−i_ refers to the feature matrix from the preceding CSF layer, and C_x_ signifies the feature matrix generated by fusing the current CSF layer.

The self-attention mechanism in the global feature block captures both spatial and temporal global information to some extent. To further enhance this, the CSF inputs the global features into the SE attention mechanism, which refines feature representation by learning the weights and interactions across feature channels. Simultaneously, the CBAM attention mechanism processes the detailed information, improving the representation of fine-grained features and emphasizing critical regions in the feature map. Subsequently, the outputs from each attention mechanism are fused through the feature fusion pathway, followed by an IRMLP module. The IRMLP structure is illustrated in Figure 5b. This approach effectively mitigates issues like gradient vanishing, gradient explosion, and network degradation, enabling the model to better capture both detailed and global features at each level. The mathematical formulation of the IRMLP process is provided in Equation (4):

(4) $$IRMLP(x)=f^{1\times 1}(f^{1\times 1}(f^{3\times 3}(x)+x))$$

$f^{1\times 1}$ is a convolution operation with a kernel size of 1 × 1. $f^{3\times 3}$ is a convolution operation with a kernel size of 3 × 3. The feature fusion operation is performed using the following formula:

(5) $$D_{out}=CBAM(D_{in})⨂D_{in}$$

(6) $$G_{out}=SE(G_{in})⨂G_{in}$$

(7) $${\hat{C}}_{x}=(Avgpool(f^{1\times 1}(c_{i-1})))$$

(8) $$C_{x}=IRMLP(D_{out}+G_{out}\;{+[D}_{in}{+G}_{in}+f^{1\times 1}({\hat{C}}_{x})])+{\hat{C}}_{x}$$

In this process, ⨂ denotes element-wise multiplication. $G_{out}$ is produced by combining the SE attention mechanism, is produced by combining the SE attention mechanism, while $D_{out}$ results from the CBAM attention mechanism. Additionally, ${\hat{C}}_{x}$ is obtained through downsampling in the preceding CSF. Lastly, the IRMLP module generates the features $C_{x}$.

#### 3.3.1. CBAM Attention Module

**Figure 6:**
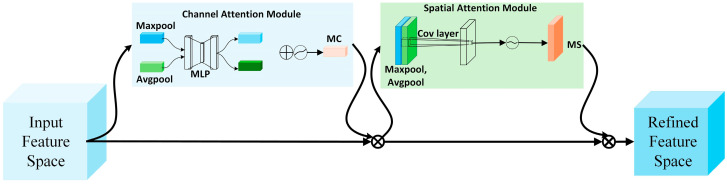
Convolutional block attention module.

CBAM [26] is a lightweight, versatile plug-and-play attention module developed to enhance the performances of CNNs. It integrates the Channel Attention Module (CAM) and the Spatial Attention Module (SAM) by concatenating them, allowing the network to focus on important features in both the spatial and channel dimensions. This approach improves feature extraction and optimizes the feature space. Figure 6 illustrates the CBAM attention module.

The Channel Attention Module (CAM) is designed to capture global dependencies across channels and dynamically adjust their significance, thereby enhancing feature representation. It comprises a global average pooling layer, a global max pooling layer, and an MLP with two fully connected layers. Initially, the input feature map undergoes both max and average pooling operations to extract key statistical information. These pooled features are then fed into the MLP, which learns channel-wise weights. The computed attention weights are subsequently applied to the original feature map, selectively amplifying or suppressing features based on their learned relevance. The mathematical formulation of CAM is presented in Equations (9) and (10). By refining channel importance, CAM enables the network to concentrate on critical image features, improving overall feature representation.

(9) $$M_{C}(F)=\sigma (MLP(Avgpool(F)))+(MLP(Maxpool(F)))$$

(10) $$F^{\prime }=M_{C}(F)\times F$$

F represents the input features; Avgpool and Maxpool represent global average pooling and global maximum pooling, respectively; MLP denotes the two fully connected layers in the multilayer perceptron; σ is the Sigmoid activation function; $M_{C}$ refers to the channel attention module; and $F^{\prime }$ represents the features obtained after passing through the channel attention module.

The Spatial Attention Module (SAM) is designed to capture spatial relationships, allowing the model to focus on essential regions within an image. It comprises a 7 × 7 convolutional layer, global average and max pooling layers, and an MLP with two fully connected layers. Initially, the input feature map undergoes global average and max pooling, and the resulting features are concatenated along the channel dimension. A convolutional layer then reduces dimensionality while extracting spatial information. The processed features are subsequently fed into the MLP, which consists of two fully connected layers. Finally, the computed spatial attention weights are applied to the original feature map, generating a refined representation. The mathematical formulation of this process is provided in Equations (11) and (12). By adaptively adjusting spatial importance, SAM enables the network to focus on key regions, enhancing overall feature representation.

(11) $$M_{S}(F^{\prime })=\sigma (f^{7\times 7}([Avgpool(F^{\prime });Maxpool(F^{\prime })]))$$

(12) $$F^{\prime \prime }=M_{S}(F^{\prime })\times F^{\prime }$$

$M_{S}$ refers to the spatial attention module; $f^{7\times 7}$ denotes the 7 × 7 convolution operation; $F^{\prime \prime }$ denotes the features obtained after the spatial attention module.

The CBAM attention mechanism combines both channel and spatial attention, allowing it to capture global and local relationships in image features. By dynamically adjusting the importance of different channels and spatial positions, CBAM enhances the extraction and utilization of key features, improving the network’s representational power and generalization. Additionally, CBAM is highly compatible with existing CNN architectures, making it easy to integrate into various network structures. It offers a simple and effective approach to enhance the performance of deep learning models.

#### 3.3.2. SE Attention Module

**Figure 7:**
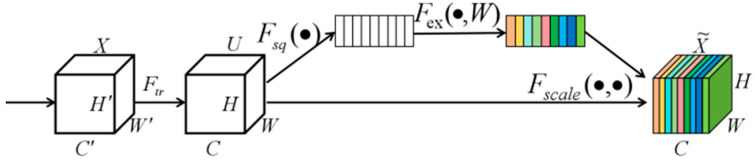
SE convolutional block attention module.

The Squeeze-and-Excitation (SE) attention module [27] is designed to boost the expressive power of convolutional neural networks (CNNs) by refining the network’s focus on various channels, thereby enhancing feature representation. This mechanism autonomously learns to assign appropriate weights to input features, enabling the model to better capture crucial information and improve its performance. The SE attention module is illustrated in Figure 7.

The input feature map is denoted as ${X\in R}^{C^{\prime }\times H^{\prime }\times W^{\prime }}$, where $C^{\prime },H^{\prime },W^{\prime }$ represent the number of channels, height, and width, respectively. Through a forward convolution operation $F_{tr}$, the feature map is transformed into ${U\in R}^{C\times H\times W}$. Next, a squeeze operation $F_{sq}(\cdot )$, a global average pooling layer, is applied to compress the spatial dimensions of each channel, resulting in a channel descriptor vector. This vector is then passed through an excitation operation $F_{ex}(\cdot ,W)$, which consists of a set of fully connected layers to generate the channel attention weights. Finally, a scaling operation $F_{scale}(\cdot ,\cdot )$ is used to apply the generated weights to the original feature map U, producing the final weighted output feature map.

The Squeeze-and-Excitation (SE) attention mechanism aims to enhance feature representation by compressing global feature information and adaptively reweighting channels. The process consists of two main steps: Squeeze and Excitation. In the Squeeze step, global average pooling is applied to the input feature map, converting each channel’s feature map into a single vector. In the Excitation step, a two-layer fully connected network is used—first reducing dimensionality and then restoring it—and a sigmoid function computes weights mapped between 0 and 1. These weights are then applied channel-wise to the original feature map, producing a reweighted feature map. Unlike traditional attention mechanisms that focus on spatial information, SE emphasizes inter-channel dependencies, thereby improving feature representation.

## 4. Experiments and Results

### 4.1. Experimental System Setup

To assess the model’s classification performance, the BreakHis and BACH datasets are utilized, both derived from histopathological images. This dataset variation helps evaluate the model’s robustness across different conditions. The data is split as follows: 80% is allocated for training, while the remaining 20% is divided equally, with 10% used for validation and 10% for testing. Table 3 and Table 4 provide a detailed breakdown of the sample distribution across these sets for both datasets. The implementation is based on PyTorch 1.13.1+cu116 and trained on an NVIDIA RTX 2080 Ti GPU with 11 GB of memory. Training is conducted over 100 epochs with a batch size of 8 and a base learning rate of 1 × 10^−4^, using a cosine annealing learning rate strategy. Input images are resized to 224 × 224, and the model employs softmax as the output activation function. The AdamW optimizer is used for optimization, while categorical cross-entropy loss is applied for loss computation.

### 4.2. Evaluation Indicators

Accuracy, F1 score, precision, and recall were chosen as classification metrics, along with the Receiver Operating Characteristic (ROC), curve to evaluate the model’s performance. These metrics are derived from the confusion matrix, as shown in Table 5, with the following definitions: True Positive (TP) represents malignant samples correctly identified as malignant, while True Negative (TN) refers to benign samples accurately classified as benign. False Positive (FP) occurs when benign samples are mistakenly predicted as malignant, whereas False Negative (FN) refers to malignant cases misclassified as benign.

Accuracy was calculated using Equation (13) to determine the proportion of correctly classified samples:

(13) $$Accuracy=\frac{TP+TN}{TP+FP+TN+FN}$$

Precision, as computed in Equation (14), indicates the proportion of true positive predictions among all predicted positives, highlighting the accuracy of the model’s positive classifications:

(14) $$Precision=\frac{TP}{TP+FP}$$

Recall, defined in Equation (15), measures the proportion of correctly identified positive samples out of all actual positive samples, reflecting the model’s ability to detect positive instances:

(15) $$Recall=\frac{TP}{TP+FN}$$

The F1-score for each category is defined in Equation (16), which balances precision and recall, with higher values indicating better performance:

(16) $$F1-score=2\frac{Precision\times Recall}{Precision+Recal}=\frac{2TP}{2TP+FP+FN}$$

In the ROC curve, the horizontal axis corresponds to the False Positive Rate (FPR), while the vertical axis represents the True Positive Rate (TPR). The False Positive Rate is the proportion of actual negative samples incorrectly classified as positive, and the True Positive Rate is the proportion of actual positive samples correctly identified as positive, as described in Equations (17) and (18):

(17) $$FPR=\frac{FP}{FP+TN}$$

(18) $$TPR=\frac{TP}{TP+FN}$$

### 4.3. MFF-ClassificationNet Model Evaluation

Table 6 presents the evaluation metrics for benign and malignant samples at different magnification levels in the BreakHis dataset. To ensure the reliability of the results, five independent data-splitting experiments were conducted, and the average values with standard deviations (±) were reported. The 95% confidence intervals from five-fold cross-validation show that the model is highly stable at 40× (98.13–98.47%) and 100× (97.26–97.96%), while slightly more variability is observed at 200× (98.25–99.38%) and 400× (95.42–96.73%), indicating higher reliability at lower magnifications and slightly lower stability at higher magnifications. The results indicate that the model performs slightly better on malignant samples, which is related to both the larger number of training images and the more distinctive structural features of malignant cells. At 40×, the accuracy for malignant samples reaches 98.98%, 2.15% higher than that of benign samples; at 100×, the difference is small; at 200× and 400×, malignant samples exceed benign samples by 1.05% and 4.11%, respectively, demonstrating the model’s advantage in recognizing malignant cells. The overall F1 scores are above 97% across all magnifications, indicating stable classification performance. Overall, malignant samples outperform benign ones across all metrics, demonstrating that MFF-ClassificationNet is robust to imbalanced datasets and effectively captures key features to enhance classification performance for malignant cases. Future comparative experiments will be conducted based on these averaged metrics to ensure consistent evaluation.

**Figure 8:**
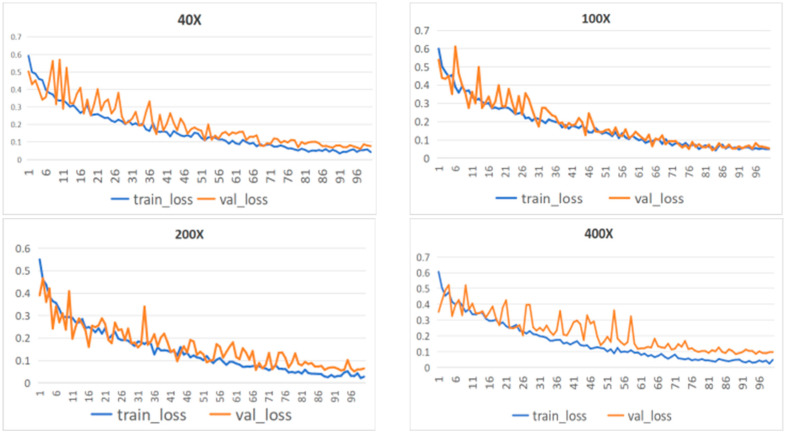
The loss curves of the MFF-ClassificationNet model on training and validation datasets with different magnifications of BreakHis.

To further evaluate the training stability of the model, Figure 8 presents the loss curves for both the training and validation sets throughout the training process. By plotting the validation loss (val_loss) and training loss (train_loss), the convergence and learning effectiveness of the model can be observed. A greater overlap between the two curves indicates more stable training and better convergence. The results show that the model exhibits a stable convergence trend across datasets at four different magnification levels. Notably, for the 100× magnification dataset, after 70 epochs, the training and validation loss curves nearly coincide, demonstrating the model’s strong learning capability. These results indicate that the model can effectively capture key abstract features from breast histopathological images while avoiding overfitting, thereby supporting the reliability of its subsequent predictive performance.

**Figure 9:**
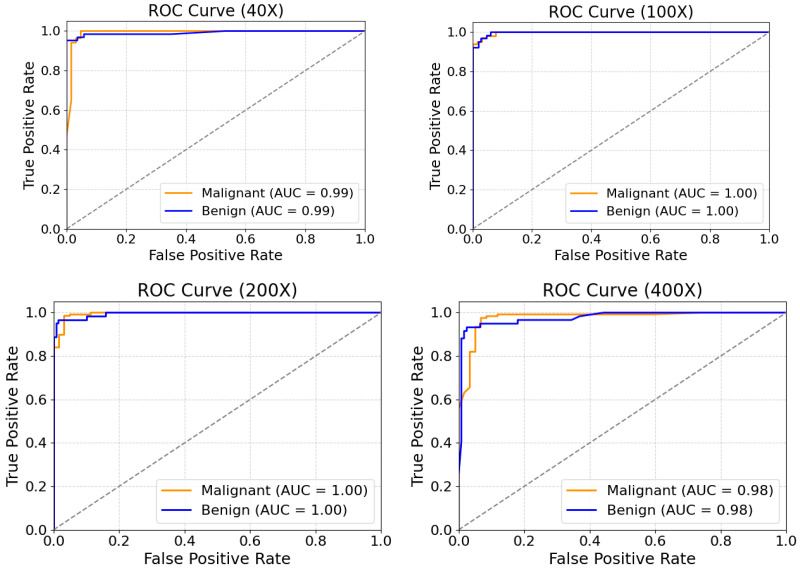
Benign and malignant ROC curves for the MFF-ClassificationNet model at different magnifications of the BreakHis test dataset.

Figure 9 presents the ROC curves of the proposed model at various magnification levels. The results indicate that at 100× and 200× magnifications, both malignant and benign categories reach a perfect average Area Under the Curve (AUC) value of 1.00. At the 40× magnification, the average AUC for both categories is 0.99, while at 400× magnification, it slightly decreases to 0.98. These findings highlight the exceptional performance of the MFF-ClassificationNet model across different magnifications, further showcasing its robust classification capability.

**Figure 10:**
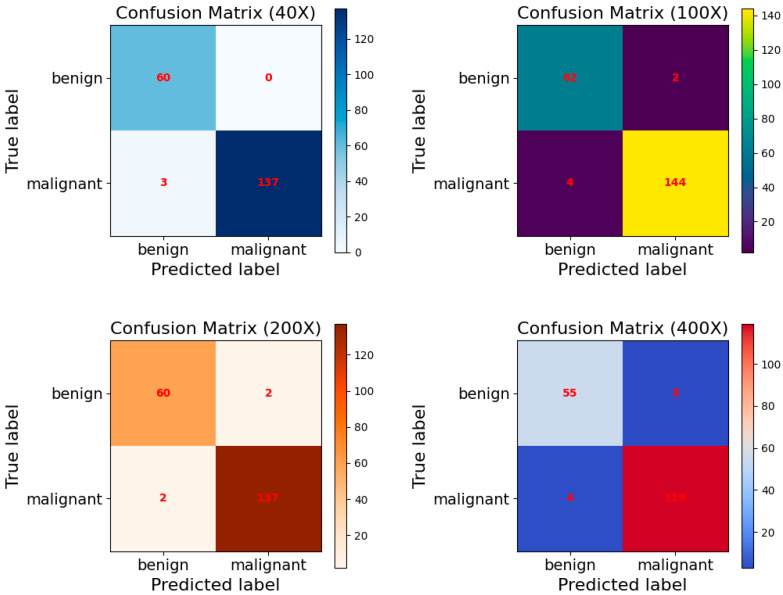
Confusion matrixes of the MFF-ClassificationNet model at different magnifications for the BreakHis test dataset.

Subsequently, by analyzing the confusion matrix of one representative test set selected from the five independent experiments (as shown in Figure 10), the correct and incorrect classifications for each category can be visually observed. At the 40× magnification level, this specific test set contains 63 benign samples and 137 malignant samples. The confusion matrix shows that 60 benign samples were correctly classified, while 3 were misclassified, resulting in a classification accuracy of 95.23%. Meanwhile, all 137 malignant samples were correctly classified, achieving a 100% accuracy rate. This result indicates that the model exhibits a stronger ability to recognize malignant cells at this magnification level. At 100×, 200×, and 400× magnifications, the model demonstrates more balanced classification performance, maintaining stable recognition capabilities for both benign and malignant samples. Overall, the proposed model exhibits consistently balanced and stable performance across different magnification levels.

### 4.4. Comparison Experiments

#### 4.4.1. Comparison of Classification Performance of Different CNNs

To verify the classification performance of MFF-ClassificationNet, classification accuracy was evaluated on the test data at four different magnifications in the BreakHis dataset. Several typical neural networks were selected as baseline models for comparison, including ResNet18, ResNet34, and ResNet50 [28], as well as deep neural networks such as DenseNet121 [15] and VGG19 [13]. Additionally, lightweight convolutional neural networks, including MobileNetV2 [29] and ShuffleNet [30], were also considered as comparison algorithms. To ensure the reliability of the results, each model was trained for 100 epochs. A thorough evaluation was carried out on all models across the four magnification levels of the BreakHis dataset, with the results summarized in Table 7. Comparing the classification accuracy of these models at various magnifications allows for a clearer assessment of the MFF-ClassificationNet model’s performance advantages.

**Figure 11:**
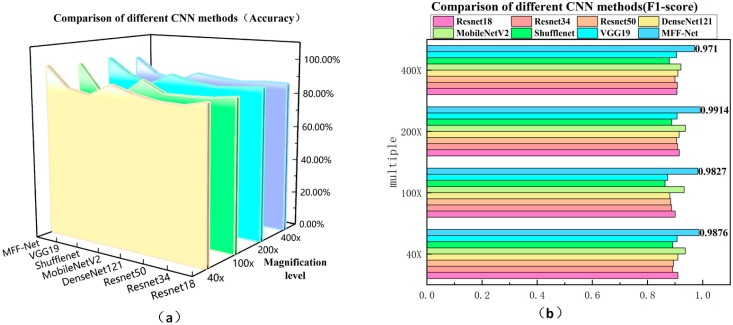
Metric graphs of different CNN methods on the BreakHis dataset: (**a**) Accuracy metrics graph of different CNN methods; (**b**) F1-score metrics graph of different CNN methods.

The experimental results in Table 7 show that the MFF-ClassificationNet model achieves outstanding predictive performance on the BreakHis dataset. At magnification levels of 40×, 100×, 200×, and 400×, MFF-ClassificationNet attains classification accuracies of 98.30%, 97.62%, 98.81%, and 96.07%, respectively. These results represent a substantial improvement over other backbone network models, highlighting the superiority of MFF-ClassificationNet in classifying breast histopathological images. To present the comparative results from Table 7 more clearly, two key metrics—Accuracy and F1-score—were selected and visualized graphically. This approach offers a more intuitive view of the trends and variations in these metrics under different experimental conditions. The resulting charts, shown in Figure 11.

Figure 11a,b compare the classification accuracy and F1-score of MFF-ClassificationNet with various CNN methods at four different magnifications. The results show that MFF-ClassificationNet consistently surpasses other CNN methods in both Accuracy and F1-score across all magnifications, highlighting its superior performance stability under varying magnification conditions.

The findings indicate that MFF-ClassificationNet is a highly efficient model for breast histopathological image classification. Its unique architecture, which includes a larger receptive field and greater sensitivity to channel information, enables it to more accurately identify critical features within images. This enhanced capability allows MFF-ClassificationNet to effectively capture key details in complex pathological images, leading to more precise classification and prediction.

#### 4.4.2. Advanced Comparison of MFF-ClassificationNet Models

To further validate the superiority of the MFF-ClassificationNet model, we conducted extensive comparative experiments involving several state-of-the-art models proposed in recent years, including Swin Transformer [25], DRDA-Net [20], VIT [31], IDsNet [32], Modified GoogLeNet [33], BMEA-ViT [34], and MobileViT [34]. These models are all improvements based on convolutional neural network (CNN) backbones or CNN-Transformer hybrid architectures, aiming to enhance the accuracy, robustness, and generalization capability of breast histopathological image classification. For a comprehensive evaluation, all models were trained and tested on the BreakHis dataset across four different magnifications (40×, 100×, 200×, 400×). The experimental results are summarized in Table 8, which show that MFF-ClassificationNet demonstrates clear advantages in multi-scale and multi-feature representations, further validating its excellent predictive performance and robustness.

Based on the experimental results presented in Table 8, the proposed MFF-ClassificationNet demonstrates excellent classification performance across four different magnification levels (40×, 100×, 200×, and 400×). At 40× magnification, the model achieves 98.30% accuracy, 98.55% precision, 98.98% recall, and 98.76% F1-score, ranking first among all comparison models. At 100× magnification, the model maintains outstanding stability, with an accuracy of 97.62% and an F1-score of 98.27%, demonstrating strong baseline classification capability. As the magnification increases to 200×, the model’s overall performance further improves, with all evaluation metrics approaching 99%, indicating that MFF-ClassificationNet can effectively capture key pathological features at different scales and exhibits strong multi-scale feature fusion and discriminative ability. At 400× magnification, although the model’s accuracy is slightly lower than some other models (such as VIT, Modified GoogLeNet, and BMEA-ViT), it still achieves superior precision, recall, and F1-score, reflecting higher classification stability and consistency. The slight decrease in accuracy at this magnification may be attributed to the reduced field of view and the more complex and localized cellular and tissue structures, making the model more sensitive to tissue heterogeneity and image noise, thereby increasing classification difficulty. Overall, MFF-ClassificationNet demonstrates excellent robustness and generalization across different magnifications, fully validating its effectiveness and reliability in multi-magnification breast histopathological image classification tasks. In comparison, other models such as DRDA-Net, VIT, Modified GoogLeNet, and BMEA-ViT, although performing well at certain magnifications, still fall short in overall stability and peak performance.

According to the experimental results shown in the table, the proposed MFF-ClassificationNet demonstrates outstanding classification performance across four different magnification levels (40×, 100×, 200×, and 400×). At 40× magnification, the model achieves 98.30% accuracy, 98.55% precision, 98.98% recall, and 98.76% F1-score, ranking first among all comparison models. At 100× magnification, the model maintains excellent stability, achieving an accuracy of 97.62% and an F1-score of 98.27%. When the magnification increases to 200×, the overall performance of the model further improves, with all four metrics approaching 99%, indicating that MFF-ClassificationNet can effectively capture key pathological features at different scales and possesses strong multi-scale feature fusion and discrimination capabilities.

It is worth noting that at 400× magnification, although the accuracy of MFF-ClassificationNet (96.07%) is slightly lower than that of the BMEA-ViT model, it achieves better results in the other three metrics—precision, recall, and F1-score—demonstrating higher stability and classification consistency. The slight decrease in accuracy at this magnification may be attributed to the narrower field of view in 400× pathological images, where cellular and tissue structures become more complex and localized, making the model more susceptible to tissue heterogeneity and image noise, thereby increasing the difficulty of discrimination.

Overall, MFF-ClassificationNet exhibits excellent robustness and generalization ability across different magnification levels, fully validating its effectiveness and reliability in multi-magnification breast histopathological image classification tasks. In comparison, other models such as DRDA-Net, BreastNet, ResNet50-CBAM, and BMEA-ViT also perform well at certain magnifications, but their overall stability and peak performance remain inferior to the proposed method.

**Figure 12:**
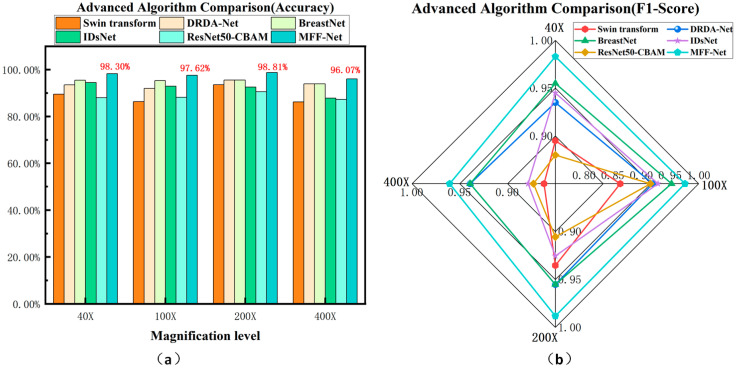
Metric plots for different models on the BreakHis dataset: (**a**) Accuracy metric plot for different models; (**b**) F1-score metric plot for different models.

F1-score, which balances precision and recall, offers a comprehensive evaluation of a model’s performance, particularly for imbalanced datasets. To better visualize performance variations across different models and conditions, the Accuracy and F1-score results from Table 8 were transformed into graphical representations, as shown in Figure 12.

Figure 12a,b compare the classification accuracy and F1-score of several advanced algorithms across four magnifications. The results clearly show that MFF-ClassificationNet consistently achieves the highest Accuracy and F1-score at all magnifications. This demonstrates its notable performance advantage over other algorithms, ensuring more precise image classification while effectively maintaining a balance between precision and recall.

**Figure 13:**
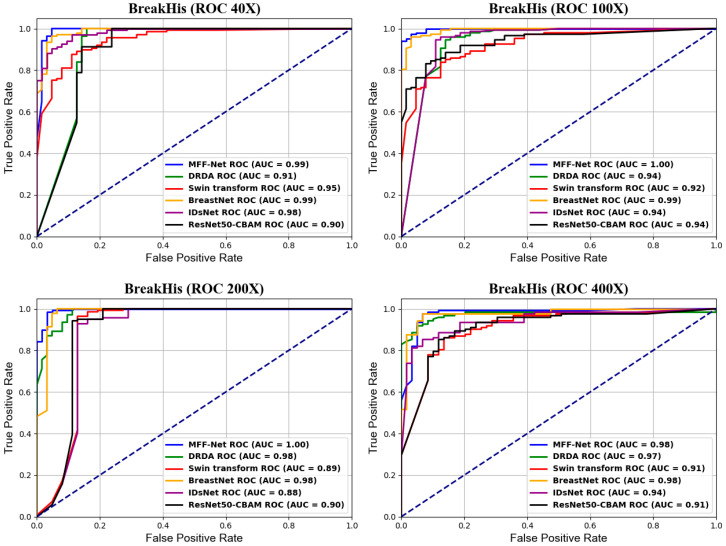
ROC curves of the MFF-ClassificationNet model with different state-of-the-art models on the BreakHis dataset.

Figure 13 presents the ROC curve comparisons of various advanced models on the BreakHis dataset at different magnifications. The ROC curve is a crucial tool for evaluating classification models, providing a clear depiction of the trade-off between sensitivity and specificity. By examining the ROC curve, one can assess a model’s performance, with curves nearer the top-left corner indicating higher accuracy. In Figure 12., the comparisons of ROC curves clearly illustrate the performance differences among the models at each magnification level.

From the ROC curve comparisons in Figure 13, it can be observed that the ROC curve of the MFF-ClassificationNet model tends to be closer to the top-left corner for most magnification levels, with its AUC values approaching 1. This indicates that MFF-ClassificationNet exhibits superior classification performance at these magnifications. Specifically, at 40× and 400× magnifications, both the MFF-ClassificationNet and BreastNet models achieve high AUC values, demonstrating strong classification capabilities at these magnifications. However, at 100× and 200× magnifications, the AUC value of MFF-ClassificationNet reaches 1.0, meaning that it can perfectly distinguish between malignant and benign cases, outperforming other advanced models.

In summary, MFF-ClassificationNet significantly outperforms other comparison models across multiple key performance metrics. Its unique multi-feature fusion architecture effectively balances the variations among images at different magnifications, ensuring more stable performance under diverse conditions and significantly enhancing overall classification accuracy. These results demonstrate that MFF-ClassificationNet possesses strong learning ability and adaptability in breast histopathological image classification tasks, allowing it to more accurately distinguish between benign and malignant tissues. Furthermore, compared to other convolutional neural network models and state-of-the-art methods, MFF-ClassificationNet not only achieves superior accuracy but also exhibits a stronger ability to capture complex image features. This makes MFF-ClassificationNet highly efficient for breast histopathological image classification.

### 4.5. Generalization Experiments

To further evaluate the generalization capability of the proposed MFF-ClassificationNet model across different datasets, additional experiments were conducted on the BACH dataset. It should be noted that the BACH dataset is independent of the BreakHis dataset. Therefore, this section focuses on assessing the model’s robustness and transferability across different data sources. The experimental results on the BACH dataset were compared with several state-of-the-art methods, including CNN + SVM, Inception V4 [35], VGG16 [14], Inception-ResNet V2 [36], EfficientNet-B0 (transformer decoder-based fusion) [37], and DWNAT-Net [38]. The classification accuracies of all models are summarized in Table 9. The results demonstrate that MFF-ClassificationNet not only achieves outstanding classification performance on the BreakHis dataset but also exhibits excellent generalization and stability across different breast histopathological image datasets.

The experimental results demonstrate that the MFF-ClassificationNet model achieved the highest predictive performance on the BACH dataset, with an accuracy of 97.50%. This represents a notable improvement over other backbone network models, further confirming MFF-ClassificationNet’s strong generalization ability across different datasets. The success of MFF-ClassificationNet can be attributed to the effective integration of its parallel branch structure and attention mechanism, which significantly enhance the network’s feature extraction capability. The parallel branch design allows the model to process multiple types of feature information simultaneously, while the attention mechanism directs the model’s focus to key features, improving classification accuracy. These traits enable MFF-ClassificationNet to effectively recognize and classify complex breast histopathological images, resulting in its exceptional performance on the BACH dataset.

**Figure 14:**
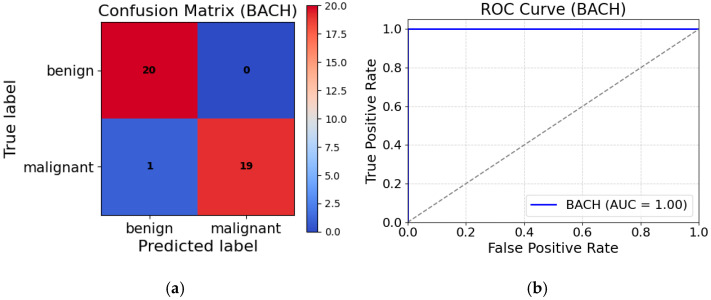
(**a**) Confusion matrix plot of MFF-ClassificationNet model on BACH test dataset; (**b**) ROC curve of MFF-ClassificationNet model on BACH test dataset.

To further evaluate the performance of the MFF-ClassificationNet model, a comprehensive analysis was conducted using its confusion matrix and ROC curve on the BACH dataset. Figure 14a presents the confusion matrix for the test dataset, which includes 20 benign and 20 malignant samples. The matrix shows that the model correctly classified all benign samples, achieving a perfect accuracy of 100% for this category. Only one malignant sample was misclassified, highlighting MFF-ClassificationNet’s ability to accurately identify benign cells while maintaining strong performance in detecting malignant samples. Additionally, the AUC value of the MFF-ClassificationNet model on the BACH dataset was computed, and the corresponding ROC curve is shown in Figure 14b.

From Figure 14b, it can be observed that the ROC curve and AUC value of the MFF-ClassificationNet model on the BACH dataset further validate its outstanding performance in breast histopathological image classification. The AUC value obtained from testing the MFF-ClassificationNett model on the BACH dataset reaches 1.00, indicating that the model achieves perfect classification on this dataset, accurately distinguishing between benign and malignant samples. This result further confirms that the MFF-ClassificationNet model can effectively extract deep features from breast histopathological images, distinguishing benign from malignant data with high precision. Its powerful feature extraction capability enables the model to accurately identify key features even in complex pathological images, leading to higher classification accuracy.

### 4.6. Visualization

To evaluate the interpretability of the MFF-ClassificationNet model and demonstrate its ability to extract meaningful features from medical images, we conducted a visualization study of the model’s learning process. This experiment employed Grad-CAM (Gradient-weighted Class Activation Mapping) [39], a widely used technique for explaining the decision-making process of convolutional neural networks. Grad-CAM identifies the regions most important for classification by computing the gradient of the classification score with respect to the feature maps in the final convolutional layer, thereby revealing the key areas the model focuses on. In this study, we visualized the final convolutional layer (excluding the linear layer) of the MFF-ClassificationNet model to highlight the regions of interest during training and generated corresponding heatmaps. Specifically, breast histopathological images at four different magnifications from the BreakHis dataset, as well as benign and malignant samples from the BACH dataset, were analyzed. The visualization results are presented in Table 10 and Table 11, clearly demonstrating that the model can effectively capture key pathological features and provide intuitive explanations, offering potential support for clinical decision-making and lesion localization. Furthermore, this analysis confirms the model’s ability to attend to multi-scale and multi-type pathological images, providing important references for subsequent model optimization and performance evaluation.

From Table 10, it can be observed that at the 40× magnification of the BreakHis dataset, the model demonstrates excellent recognition capability for malignant cases. The heatmaps generated by Grad-CAM clearly highlight the model’s strong focus on malignant lesion areas when processing pathological images, further verifying its precise localization ability for malignant regions at this magnification. At 100× magnification, the Grad-CAM-generated heatmap covers a more dispersed area but still effectively identifies key features of the lesion region. At 200× and 400× magnifications, the heatmaps exhibit a clearer focus on highly relevant lesion areas in the pathological images, showcasing the model’s stable ability to recognize lesions across different magnifications. This visualization analysis not only aids in understanding the model’s performance across different magnifications but also provides valuable insights for further optimizing its performance.

From Table 11 it can be observed that in the BACH dataset, the model demonstrates strong recognition capabilities for both benign and malignant cases. The heatmaps generated by Grad-CAM accurately highlight the lesion characteristics, indicating that the MFF-ClassificationNet model has high precision in localizing lesion areas and can effectively identify disease-related regions. This capability is crucial for the early diagnosis and treatment of breast cancer, as it assists doctors in quickly and accurately identifying lesion areas, thereby improving diagnostic accuracy and efficiency.

In conclusion, the MFF-ClassificationNet model greatly improves feature extraction by combining parallel branches with attention mechanisms. It shows significant strengths in breast histopathological image classification, offering valuable technical support for medical diagnosis. Additionally, the model’s consistent performance across various magnifications highlights its reliability and robustness in handling pathological images, making it highly applicable to real-world clinical settings.

### 4.7. Ablation Experiments

To comprehensively evaluate the effectiveness of each component in the proposed MFF-ClassificationNet model, a series of six ablation experiments was designed and conducted. These experiments assessed the contribution of each individual feature extraction path, combinations of two modules, as well as the individual components within the Channel-Spatial Fusion (CSF) module—namely, the Convolutional Block Attention Module (CBAM) and the Squeeze-and-Excitation (SE) mechanism—to provide a clearer understanding of how each part contributes to performance improvement. Specifically, six comparative model variants were constructed: (1) a network containing only the Detail Enhancement Module (DEM); (2) a network containing only the Global Feature Module (GFM); (3) a network combining the global and detail modules (Detail and Global Fusion, DGF); (4) a network combining the global and detail modules along with a CSF module that uses only the SE mechanism (Detail, Global, and SE Fusion, DGSF); (5) a network combining the global and detail modules along with a CSF module that uses only the CBAM mechanism (Detail, Global, and CBAM Fusion, DGCF); (6) the full MFF-ClassificationNet model, which integrates the Global Feature Module, Detail Enhancement Module, and the complete CSF module that jointly incorporates both CBAM and SE mechanisms.

All models were trained under identical experimental settings, including the same loss function, optimizer, and hyperparameters, and were evaluated on the BreakHis dataset across different magnification levels. The results, presented in Table 12, compare the models based on classification accuracy. The findings confirm the effectiveness of each individual module and demonstrate the superior performance of the multi-feature fusion strategy. Notably, within the CSF module, the combined use of CBAM and SE mechanisms outperformed models using either one alone, further illustrating the benefit of integrating both channel-spatial attention and squeeze-and-excitation mechanisms to enhance feature representation capabilities.

This study systematically verifies the contribution of each module in the MFF-ClassificationNet model through a series of ablation experiments, with the results summarized in the corresponding table. The performance of individual modules was first evaluated: the model with only the Detail Enhancement Module (DEM) achieved accuracies of 95.00% and 95.50% at 40× and 200× magnifications, respectively, while the Global Feature Module (GFM) slightly outperformed DEM at 40× with an accuracy of 95.50%. When DEM and GFM were combined into the DGF model, performance improved significantly, especially at 200× magnification where it reached 97.50% accuracy. Further incorporating the SE mechanism, the DGSF model demonstrated outstanding performance at 100× and 400× magnifications, achieving 96.00% and 95.58% accuracy, respectively. Meanwhile, the DGCF model, which integrates DEM, GFM, and the CBAM mechanism, maintained stable results at 40× and 100× magnifications, with accuracies of 96.50% and 95.75%, respectively. Finally, the complete MFF-ClassificationNet, which integrates DEM, GFM, CBAM, and SE modules, achieved the best performance across all magnification levels. Notably, it reached 98.30% and 98.81% accuracy at 40× and 200×, significantly surpassing other model configurations. These results clearly demonstrate the effectiveness of the multi-feature fusion strategy and highlight the importance of the synergy among modules in improving model performance. The steady increase in accuracy with the progressive addition of modules further validates the design value of each component and the rationality of the overall integration strategy.

The MFF-ClassificationNet model leverages a combination of the global feature module, detail enhancement module, and CSF module, allowing for better integration of multi-scale features and attention mechanisms. This significantly boosts its ability to classify breast tissue pathology images. The multi-feature fusion strategy not only improves the model’s focus on lesion areas but also enhances its robustness and accuracy across various magnifications. Additionally, the diverse and complex nature of the BreakHis dataset underscores the performance gains achieved by MFF-ClassificationNet. The model’s impressive results on such a challenging dataset highlight its strong clinical value and generalization capability for real-world applications. In conclusion, the MFF-ClassificationNet model offers substantial performance improvements in breast pathology image classification, thanks to its multi-feature fusion approach, providing a more accurate and reliable tool for computer-aided breast cancer diagnosis.

## 5. Discussion

This study presents MFF-ClassificationNet and its multi-feature fusion design for breast histopathological image classification. The network employs a dual-branch architecture that simultaneously extracts local fine-grained features and global contextual information, enabling hierarchical and multi-scale representation of breast tissue images. This design allows the model to capture subtle pathological patterns while preserving the overall tissue structure, which is particularly important for highly heterogeneous and complex histopathological images. By effectively integrating features across different scales and types, the model can fully exploit both the rich details and global organization present in pathological images, thereby enhancing classification performance and diagnostic reliability.

Ablation studies and Grad-CAM visualizations indicate that the network effectively attends to key pathological regions, enhancing interpretability and providing valuable support for clinical lesion localization. Specifically, Grad-CAM heatmaps demonstrate that the model can accurately identify suspicious tumor regions even in cases with subtle morphological changes, highlighting its potential to assist pathologists in decision-making. Moreover, the multi-feature, multi-scale representation provides intuitive insights into complex spatial patterns in pathology images, helping clinicians better understand the basis of the model’s predictions and increasing trust in its outputs.

To highlight the potential clinical utility of MFF-ClassificationNet, the model can support practical diagnostic workflows by assisting pathologists in identifying suspicious tumor regions, reducing oversight and improving diagnostic accuracy; providing rapid preliminary assessments to prioritize cases requiring urgent attention; and capturing both local fine-grained features and global tissue information through its dual-branch architecture, thereby supporting multi-scale lesion localization for comprehensive pathological evaluation. Regarding interpretability and validation, ablation studies and Grad-CAM visualizations confirm that the model effectively focuses on critical pathological regions, offering spatial insights that enhance clinical trust. It should be noted that, although Grad-CAM provides some interpretability, other feature attribution methods such as SHAP were not used in this study. This was primarily due to SHAP’s high computational cost and implementation complexity, making large-scale application impractical under the current experimental conditions. Nevertheless, SHAP and similar methods have been identified as a future research direction to further enhance the transparency and clinical utility of model predictions.

In terms of computational complexity, the model has 18.52 GMac FLOPs, 125.53 million parameters, and an average inference time of 27.25 ms per image on GPU. While this scale enables rich feature extraction and high classification accuracy, the computational overhead may limit deployment in resource-constrained clinical environments. To improve deployability and practical applicability, lightweight network designs could be implemented to achieve faster inference, making the model suitable for low-resource clinical settings.

Despite strong generalization across datasets, limitations remain. In particular, when applied to the BACH dataset, the model’s performance may be affected by the relatively small sample size, class imbalance, and variations in staining and imaging protocols. Moreover, the model primarily relies on image features without integrating clinical metadata (e.g., patient history or biomarkers), which could further improve diagnostic accuracy.

Based on these limitations, future work will focus on the following directions: model lightweighting through developing more efficient architectures or pruning strategies to reduce computational costs while maintaining classification performance; multi-modal fusion, integrating complementary information from different imaging modalities (e.g., immunohistochemistry, radiology) and other relevant data to enhance robustness and diagnostic accuracy; cross-dataset transfer learning, leveraging multiple datasets to improve generalization across diverse populations, imaging devices, and conditions; incorporation of clinical metadata, combining patient demographics, history, and biomarker information for more comprehensive diagnostic decision support; and explainability enhancement, further refining interpretability mechanisms (including planned exploration of SHAP or similar methods) to provide transparent and clinically actionable insights from model predictions.

## 6. Conclusions

This study proposes MFF-ClassificationNet for the classification of breast histopathological images, implemented with an end-to-end training strategy in the PyTorch framework. By effectively combining the strengths of CNNs and Transformers, MFF-ClassificationNet significantly improves classification accuracy and robustness. The network adopts a dual-branch parallel structure and integrates the CSF module to enhance feature learning, enabling simultaneous extraction of local fine-grained features and global contextual information. This design facilitates multi-scale, hierarchical representation of breast tissue images, allowing the model to capture subtle pathological patterns while preserving overall tissue structure, thereby improving classification performance for complex pathological images.

On the BreakHis dataset at different magnification levels, the model achieves classification accuracies of 98.30%, 97.62%, 98.81%, and 96.07%, demonstrating stability and efficiency across scales. On the BACH dataset, the model attains an accuracy of 97.50%, further validating its strong generalization capability. Ablation studies and Grad-CAM visualizations show that MFF-ClassificationNet effectively focuses on key pathological regions, extracts deep features, enhances interpretability, and provides valuable support for clinical lesion localization.

Despite these advantages, several limitations remain. First, the high computational cost during training and inference may restrict deployment in resource-limited environments. Second, experiments are primarily based on the BreakHis and BACH datasets, and model performance needs to be validated on larger or more diverse datasets. Additionally, although Grad-CAM provides interpretability, the clinical usability of the model’s predictions could be further improved using more advanced explanation methods. Future work will focus on model lightweighting to reduce computational costs, multi-modal information fusion to enhance robustness and diagnostic accuracy, cross-dataset transfer learning to improve generalization, and interpretability optimization to provide transparent and clinically actionable predictions.

In summary, MFF-ClassificationNet provides a robust and efficient framework for multi-scale, multi-feature analysis of breast histopathological images, showing promising potential for early breast cancer detection, auxiliary diagnosis, and the development of intelligent medical imaging systems, while offering valuable methodological guidance for future related research.

## Figures and Tables

**Table 1:** Image Distribution of the BreakHis Dataset.

Magnification Level	Benign	Malignant	Total
40×	625	1370	1995
100×	644	1437	2081
200×	623	1390	2013
400×	588	1232	1820
Total	2480	5429	7909

**Table 2:** The distribution of the BACH Dataset.

Category	Subtype	Subtype Count	Total
Benign	Normal	100	200
	Benign	100	
Malignant	InSitu Carcinoma	100	200
	InvasiveCarcinoma	100	

**Table 3:** Sample division of the BreakHis dataset.

Magnification Level	Train Set	Validation Set	Test Set
40×	1596	199	200
100×	1654	204	211
200×	1611	201	201
400×	1458	181	181

**Table 4:** Sample division of the BACH dataset.

Input Shape	Train Set	Validation Set	Test Set
(224, 224, 3)	320	40	40

**Table 5:** Dichotomous confusion matrix.

	Predicted Label Positive	Predicted Label Negative
Actual Label Positive	True Positive (TP)	False Negative (FN)
Actual Label Negative	False Positive (FP)	True Negative (TN)

**Table 6:** The performance metrics obtained by the MFF-ClassificationNet model on the BreakHis test dataset with different magnifications.

Magnification Level	Class	Accuracy	Precision	Recall	F1-Score	Support	95% CI
40×	Benign	96.83 ± 1.42%	97.80 ± 1.56%	96.83 ± 1.42%	97.29 ± 0.38%	63 ± 1	
	Malignant	98.98 ± 0.74%	98.55 ± 0.63%	98.98 ± 0.74%	98.76 ± 0.18%	137 ± 1	
	Overall (Mean ± SD)	98.30 ± 0.24%	98.55 ± 0.63%	98.98 ± 0.74%	98.76 ± 0.18%		[98.13%, 98.47%]
100×	Benign	97.22 ± 0.62%	95.19 ± 1.68%	97.22 ± 0.62%	96.19 ± 0.94%	64 ± 1	
	Malignant	97.80 ± 0.80%	98.75 ± 0.27%	97.80 ± 0.80%	98.27 ± 0.44%	148 ± 3	
	Overall (Mean ± SD)	97.62 ± 0.60%	98.75 ± 0.27%	97.80 ± 0.80%	98.27 ± 0.44%		[97.26%, 97.96%]
200×	Benign	98.08 ± 1.86%	98.09 ± 1.18%	98.08 ± 1.86%	98.08 ± 1.21%	62 ± 1	
	Malignant	99.14 ± 0.54%	99.14 ± 0.83%	99.14 ± 0.54%	99.14 ± 0.54%	139 ± 1	
	Overall (Mean ± SD)	98.81 ± 0.74%	99.14 ± 0.83%	99.14 ± 0.54%	99.14 ± 0.54%		[98.25%, 99.38%]
400×	Benign	93.30 ± 1.80%	94.57 ± 0.0122	93.30 ± 0.0180	93.91 ± 0.0101	59 ± 1	
	Malignant	97.41 ± 0.61%	96.80 ± 0.83%	97.41 ± 0.61%	97.10 ± 0.46%	122 ± 2	
	Overall (Mean ± SD)	96.07 ± 0.63%	96.80 ± 0.83%	97.41 ± 0.61%	97.10 ± 0.46%		[95.42%, 96.73%]

**Table 7:** The metrics of different CNN methods on the BreakHis dataset. Bold values indicate the best performance for each metric.

Model	Metric	Results on Different Scales of the BrakHis Dataset			
		40×	100×	200×	400×
Resnet18	Accuracy	87.00%	86.32%	88.06%	87.29%
	Precision	86.27%	91.61%	89.66%	89.60%
	Recall	96.35%	88.51%	93.53%	91.80%
	F1-Score	91.03	90.03%	91.55%	90.69%
Resnet34	Accuracy	84.00%	84.43%	87.06%	87.29%
	Precision	83.02%	89.66%	88.44%	88.37%
	Recall	96.35%	87.84%	93.53%	93.44%
	F1-Score	89.19%	88.74%	90.91%	90.84%
Resnet50	Accuracy	84.50%	83.96%	86.07%	86.19%
	Precision	83.97%	89.04%	84.91%	88.19%
	Recall	95.62%	87.84%	97.12%	91.80%
	F1-Score	89.42%	88.44%	90.60%	89.96%
DenseNet121	Accuracy	87.00%	83.96%	87.56%	87.29%
	Precision	86.27%	91.30%	87.01%	86.67%
	Recall	96.35%	85.14%	96.40%	95.90%
	F1-Score	91.03%	88.11%	91.47%	91.05%
MobileNetV2	Accuracy	91.00%	90.57%	91.04%	88.95%
	Precision	89.93%	92.67%	90.60%	88.64%
	Recall	97.81%	93.92%	97.12%	95.90%
	F1-Score	93.71%	93.29%	93.75%	92.13%
Shufflenet	Accuracy	84.00%	80.19%	84.08%	83.43%
	Precision	83.02%	83.13%	86.39%	86.51%
	Recall	96.35%	89.86%	91.37%	89.34%
	F1-Score	89.19%	86.36%	88.81%	87.90%
VGG19	Accuracy	86.50%	81.60%	89.05%	86.74%
	Precision	96.35%	90.54%	97.12%	94.26%
	Recall	96.35%	90.54%	97.12%	94.26%
	F1-Score	90.72%	87.30%	90.72%	90.55%
MFF-ClassificationNet	Accuracy	**98.30%**	**97.62%**	**98.81%**	**96.07%**
	Precision	**98.55%**	**98.75%**	**99.14%**	**96.80%**
	Recall	**98.98%**	**97.80%**	**99.14%**	**97.41%**
	F1-Score	**98.76%**	**98.27%**	**99.14%**	**97.10%**

**Table 8:** Metrics for different advanced models on the BreakHis dataset. Bold values indicate the best performance for each metric.

Factor	Metric Model	IDsNet (2021)	Swin Transform (2021)	DRDA-Net (2022)	MOBILE VIT (2024)	VIT (2024)	Modified GoogLeNet (2024)	BMEA-ViT (2025)	MFF-ClassificationNet
40×	Accuracy	94.50%	89.50%	93.50%	94.33%	96.50%	97.24%	95.74%	**98.30%**
	Precision	95.00%	89.73%	93.06%	93.34%	95.88%	96.89%	94.50%	**98.55%**
5	Recall	97.08%	95.62%	97.81%	93.34%	95.88%	96.69%	94.50%	**98.98%**
	F1-Score	96.03%	92.58%	95.37%	93.34%	96.50%	96.79%	95.00%	**98.76%**
100×	Accuracy	92.92%	86.32%	91.98%	92.00%	94.66%	96.88%	96.96%	**97.62%**
	Precision	94.63%	88.39%	93.38%	92.49%	95.18%	96.09%	96.00%	**98.75%**
	Recall	95.27%	92.57%	95.27%	92.49%	95.18%	96.67%	95.00%	**97.80%**
	F1-Score	94.95%	90.43%	94.31%	92.49%	94.66%	96.37%	95.50%	**98.27%**
200×	Accuracy	92.54%	93.53%	95.52%	96.91%	97.87%	97.77%	98.18%	**98.81%**
	Precision	94.93%	92.57%	95.14%	97.32%	98.70%	97.29%	97.50%	**99.14%**
	Recall	94.24%	98.56%	98.56%	97.32%	98.70%	97.50%	98.00%	**99.14%**
	F1-Score	94.58%	95.47%	96.82%	97.31%	97.87%	97.40%	98.00%	**99.14%**
400×	Accuracy	87.85%	86.19%	93.92%	92.03%	**98.56%**	98.08%	97.25%	96.07%
	Precision	93.10%	86.47%	95.87%	89.91%	**98.35%**	97.71%	96.50%	96.80%
	Recall	88.52%	94.26%	95.08%	89.91%	**98.35%**	97.92%	97.00%	97.41%
	F1-Score	90.76%	90.20%	95.47%	89.91%	**98.56%**	97.81%	97.00%	97.10%

**Table 9:** The accuracy results of different methods on the BACH dataset. Bold values indicate the best performance for each metric.

Model	Accuracy Results on the BACH Dataset
CNN + SVM	83.30%
Inception V4	93.70%
VGG16	93.80%
Inception-ResNet V2	93.75%
EfficientNet-B0 (transformer decoder-based fusion)	96.66
DWNAT-Net	93.75%
MFF-ClassificationNet	**97.50%**

**Table 10:** Breast histopathology images at different magnifications in the BreakHis dataset and the corresponding Grad-CAM visualization of MFF-ClassificationNet.

Image Class	BreakHis Dataset(40×)			
	Benign		Malignant	
Original images	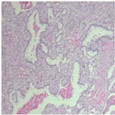	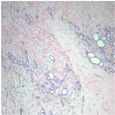	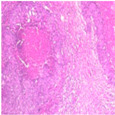	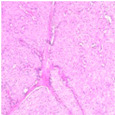
MFF-ClassificationNet heatmaps	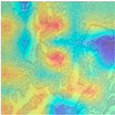	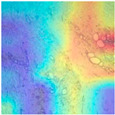	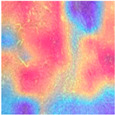	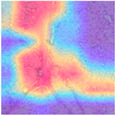
Image Class	BreakHis dataset (100×)			
	benign		malignant	
Original images	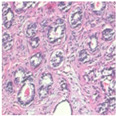	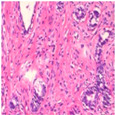	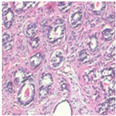	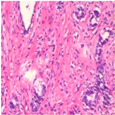
MFF-ClassificationNet heatmaps	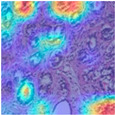	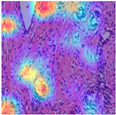	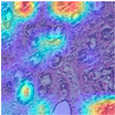	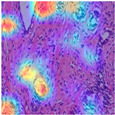
Image Class	BreakHis dataset (200×)			
	benign		malignant	
Original images	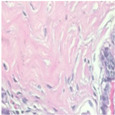	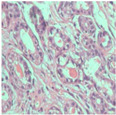	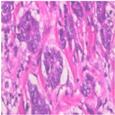	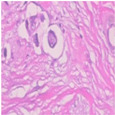
MFF-ClassificationNet heatmaps	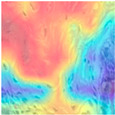	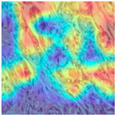	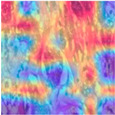	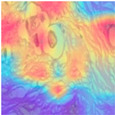
Image Class	BreakHis dataset (400×)			
	benign		malignant	
Original images	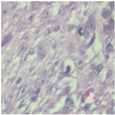	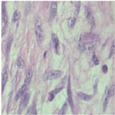	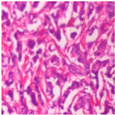	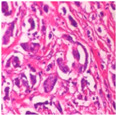
MFF-ClassificationNet heatmaps	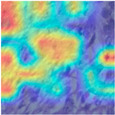	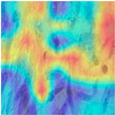	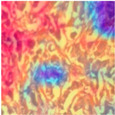	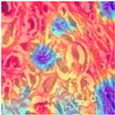

**Table 11:** Breast histopathology image of the BACH dataset and the corresponding Grad-CAM visualization of the MFF-ClassificationNet.

Image Class	BACH Dataset			
	Benign		Malignant	
Original images	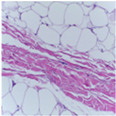	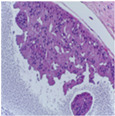	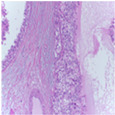	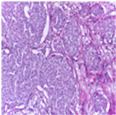
MFF-ClassificationNet heatmaps	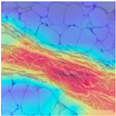	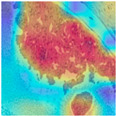	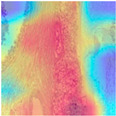	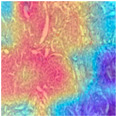

**Table 12:** The accuracy results for the four models on the BreakHis dataset. Bold values indicate the best performance for each metric.

Method	DEM	GFM	SE	CBAM	40×	100×	200×	400×
DEM	√				95.00%	92.92%	95.50%	93.37%
GFM		√			95.50%	93.80%	94.02%	92.81%
DGF	√	√			96.00%	94.33%	95.50%	94.47%
DGSF	√	√		√	96.50%	96.00%	96.02%	95.58%
DGCF	√	√	√		96.50%	95.75%	96.02%	95.10%
MFF-ClassificationNet	√	√	√	√	**98.30%**	**97.62%**	**98.81%**	**96.07%**

## Data Availability

The dataset used in this study is publicly available and can be accessed https://web.inf.ufpr.br/vri/databases/breast-cancer-histopathological-database-breakhis/ (accessed on 30 October 2024) and https://iciar2018-challenge.grand-challenge.org/Dataset/ (accessed on 15 December 2024). All relevant data for analysis can be obtained from the corresponding author upon reasonable request.
